# Mapping allergy research: A comprehensive visual and bibliometric analysis of socioeconomic and quality-of-life dimensions

**DOI:** 10.1016/j.jacig.2025.100553

**Published:** 2025-08-12

**Authors:** Polat Goktas, Nicholas L. Rider, Lavanya Diwakar

**Affiliations:** a Faculty of Engineering and Natural Sciences, Sabanci University, Istanbul, Turkey; b Department of Health Systems & Implementation Science, Virginia Tech Carilion School of Medicine, Roanoke, Va; c Department of Medicine, Section of Allergy-Immunology, The Carilion Clinic, Roanoke, Va; d University Hospital of North Midlands NHS Trust, Stoke-on-Trent, United Kingdom; e University of Birmingham, Birmingham, United Kingdom

**Keywords:** Allergy, allergic disease, bibliometric, health care, health-related quality of life, economic evaluations, VOSviewer

## Abstract

**Background:**

The increasing prevalence of allergic diseases has increased attention to their socioeconomic impact and health-related quality of life. Yet, research in this domain remains dispersed and underrepresented across disciplines and regions.

**Objective:**

We sought to analyze allergy research related to socioeconomic impacts and quality of life from 1994 to 2025 and identifying key trends, collaborations, and emerging themes.

**Methods:**

A total of 913 documents from 412 sources were retrieved from the Web of Science and Scopus databases using the Preferred Reporting Items for Systematic Reviews and Meta-Analyses flow diagram. Bibliometric tools, including VOSviewer and the Bibliometrix R package, were used to assess publication growth, authorship patterns, collaboration networks, and thematic trends.

**Results:**

The annual growth rate was 5.68%, with 6229 authors and an international coauthorship rate of 24.42%, reflecting robust global collaboration. Keyword trends revealed a shift from asthma to food allergies and patient-centered themes such as quality of life, treatment satisfaction, and digital health. Thematic evolution showed increasing interest in psychosocial care, chronic allergy conditions, and technological interventions. Although the United States and the United Kingdom remain leading contributors, research from Asia and non–English-speaking countries increased following the inclusion of non-English literature.

**Conclusions:**

Allergy research is evolving toward a more holistic, interdisciplinary, and globally engaged model. Future efforts should focus on bridging regional disparities, incorporating underrepresented disciplines, and promoting inclusive, patient-centered research.

## Graphical abstract

**Figure:**
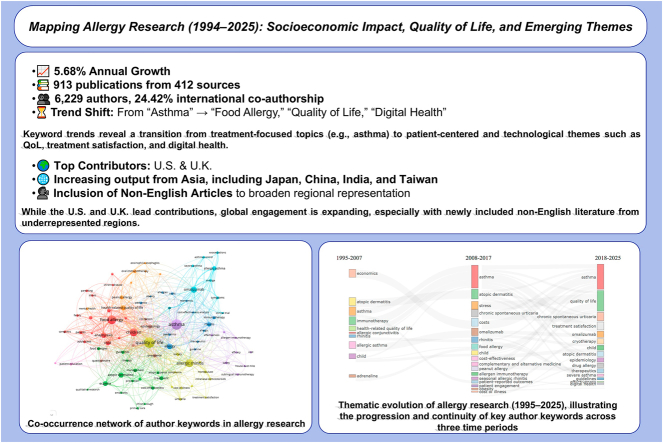

As the global prevalence of allergies rises, there is a growing focus on the associated socioeconomic impacts, quality-of-life (QoL) issues, and a deeper interest in understanding the perspectives of patients and health care providers.^1^, ^2^, ^3^ Over the past 2 decades, the management of allergic diseases has undergone a transformative shift, driven by growing awareness of the profound QoL challenges these conditions impose. There has been a transition in treatment goals for common allergic conditions, such as allergic rhinitis and food allergies, from mere symptom management and allergen avoidance to a focus on immune modulation through innovative approaches such as immunotherapy. Moreover, allergy management has embraced trends such as shared decision making with improved patient involvement, and there have been calls for more inclusive research to address the diverse experiences of individuals across varying socioeconomic and cultural contexts.^4^ Through bibliometric analysis, this article explores these evolving dimensions, offering a comprehensive overview of allergy-related research and practices from 1994 onward.

Bibliometrics serves as a powerful tool to summarize and analyze research within a specific field, enabling the identification of key trends and advancements in medical science.^5^^,^^6^ Using analytical platforms such as VOSviewer (Visualizing Scientific Landscapes),^7^^,^^8^ CiteSpace,^9^^,^^10^ and Bibliometrix,^11^ researchers can visualize and interpret data to uncover significant patterns and shifts over time. This bibliometric analysis contributes substantially to the field of allergy by providing a comprehensive overview of the health, socioeconomic, and QoL challenges associated with allergies since 1994. It highlights systemic gaps in allergy service delivery, examines the economic and psychosocial burdens experienced by individuals, and emphasizes the growing importance of patient-centered care and shared decision making in allergy management. Furthermore, it outlines future directions for research and clinical practice, aiming to address existing disparities and improve outcomes for patients. By examining these critical dimensions, this analysis advances the conversation on enhancing allergy care, steering it toward more effective, equitable, and patient-focused health care solutions.

## Methods

### Search strategy

The Web of Science and Scopus databases were used as the data sources for this study.^12^ The search strategy used keyword-based query syntax to encompass a wide range of topics related to the socioeconomic and QoL aspects of allergies. Keywords included “cost of illness,” “quality of life,” “socioeconomic factors,” and “healthcare policy,” combined with allergy-specific terms such as “allergy,” “allergic rhinitis,” “atopic dermatitis,” “asthma,” and general root terms (eg, allergy)^∗∗^. The rationale for including allergy-specific terms such as “allergic asthma” instead of broader terms such as “severe asthma” was to ensure alignment with IgE-mediated, immunologically distinct conditions frequently classified under the allergy domain. Similarly, biologic treatments such as “omalizumab” were included on the basis of their high frequency in author keyword listings across the data set and their established relevance in allergy treatment guidelines. We acknowledge that this strategy may have underrepresented non–IgE-mediated conditions or newer biologics not yet commonly indexed under allergy-focused literature. Although this approach was designed to capture broad representations of allergy-related conditions, we acknowledge that more specific terms such as “angioedema” or “eosinophil-associated diseases” (beyond eosinophilic esophagitis) may not have been explicitly included, which represents a limitation of the search strategy. The search included articles published in both English and non-English languages. Specific search strings and methodologies are documented in the Online Repository (available at www.jaci-global.org).

#### Inclusion criteria

• Studies explicitly discussing the socioeconomic impacts, health-related QoL, and psychosocial aspects of allergies.
• Publications offering insights into management strategies, health care provider experiences, patient perspectives, and policy implications related to allergies.

#### Exclusion criteria

• Articles not focusing on socioeconomic impacts or QoL in allergy and immunology.
• Publications not directly addressing the thematic scope concerning allergies’ impact on socioeconomic factors, health care systems, or individuals’ QoL.
• Publications before 1994, considering the focus on more recent developments.
• Articles without primary data or those that were not suitable for analysis using the software packages (review articles, conference abstracts, articles in press, book chapters).

### Identification and selection of documents

**Fig 1:**
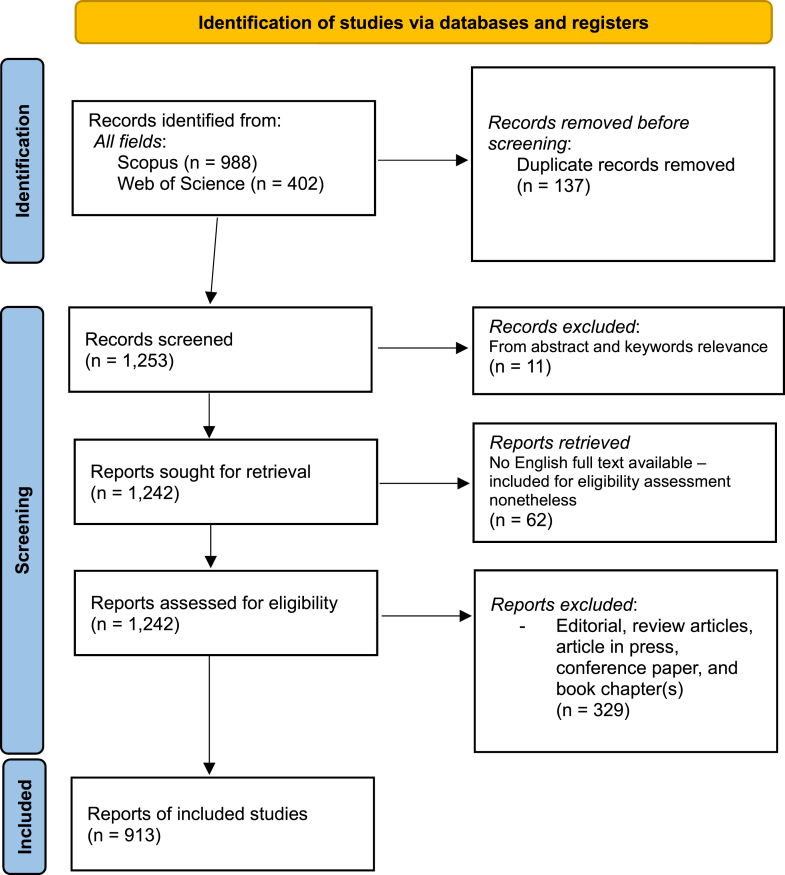
PRISMA flow diagram illustrating the document selection process for studies on the health/socioeconomic impacts and QoL in the field of allergy. *PRISMA*, Preferred Reporting Items for Systematic Reviews and Meta-Analyses.

This study followed the Preferred Reporting Items for Systematic Reviews and Meta-Analyses framework, with the scoping reviews extension by Tricco et al.^13^ Of the 1390 publications identified, 913 were eligible for analysis (see Fig 1).

### Data analysis

The bibliometric analysis used VOSviewer and the Bibliometrix R package for their respective advantages. VOSviewer (version 1.6.18) facilitates network construction and visualization on the basis of publications, countries, institutions, journals, cocited journals, authors, cocited authors, and keywords.^7^^,^^8^ In VOSviewer’s visual network diagrams, circles represent analytical items such as countries, institutions, journals, or authors, with circle size indicating the number of items and colors representing different clusters. The thickness of connecting lines reflects the degree of cooperation or co-citation among the items. The Bibliometrix R package (version 4.2.1) (https://www.bibliometrix.org) was used for comprehensive bibliometric and visual analysis. Using Bibliometrix, we constructed Bradford law diagrams, relevant sources, and trend topic analyses.^11^ By analyzing citation patterns, coauthorship networks, and keyword trends, we were able to identify key contributors, thematic areas, and emerging research directions in the field.

To categorize the evolution of research themes, a thematic map was generated, organizing key themes into 4 distinct quadrants on the basis of their centrality and density within the research field. This thematic structure helps map the dynamic nature of allergy research, identifying areas of both foundational and emerging importance.

## Results

### Descriptive insights from more than 2 decades of research in allergy

Our bibliometric analysis spans allergy research from January 1, 1994, to April 24, 2025, and we identified 913 unique publications for analysis (Fig 1). The data set, sourced from 412 journals, shows a steady annual growth rate of 5.68%, indicating sustained scholarly interest. The average document age is 10.2 years, with each document averaging 41.85 citations, underlining the research’s lasting impact. Contributions came from 6229 authors, with an average of 9.03 coauthors per document and an international coauthorship rate of 24.42%, highlighting strong global collaboration. Scientific production increased notably from 2007, peaking in 2017 with 60 articles. Citation trends reveal early articles from 1994 still average 0.4 citations per year, whereas recent 2024 publications show a citation rate of 1.05 per year, indicating early academic engagement. This comprehensive data set provides crucial insights into the evolution and impact of allergy research over nearly 3 decades, as detailed in Table I.

**Table I:** Summary of bibliographic data on allergy research (1994-2025)

Indicator	Description
Timespan	1994-2025
Sources (journals, books, etc)	412
Documents	913
Annual growth rate (%)	5.68
Average document age (y)	10.2
Average citations per document	41.85
Keywords Plus (ID)	6675
Author’s keywords (DE)	1741
Authors	6229
Authors of single-authored documents	37
Single-authored documents	37
Coauthors per document	9.03
International coaauthorships (%)	24.42

The “Timespan” indicates the period of research publication considered by the study, underscoring the extent of historical data examined.

“Sources” signify the variety of journals, books, and other mediums that have disseminated the body of work assessed, illustrating the research’s reach.

“Documents” denote the aggregate number of scholarly articles that have been analyzed, signifying the bulk of research conducted in the field.

The “Annual Growth Rate” quantifies the yearly increase in publication volume, offering insight into the expanding momentum of allergy-related research.

“Average Document Age” refers to the mean age of all documents included in the data set, calculated by subtracting the publication year of each document from the current year (2025) and averaging these values. This metric helps to assess how contemporary or outdated the analyzed literature is, thereby providing context on the timeliness and possible relevance of the findings in today’s research landscape.

“Average Citations per Document” offer a metric for the average influence or impact each document has had within the scholarly community, via citation frequency.

“Keywords Plus” and “Author’s Keywords” capture the expanse and variation in research themes addressed in the documents, indicating the breadth of the field’s exploration.

The count of “Authors” and the subset of “Authors of Single-authored Documents” depict the community size involved in the research and the instances of individual scholarly work, respectively.

“Coauthors per Document” and the percentage of “International Coauthorships” provide a window into the collaborative nature of the research efforts and the global scale of research partnerships, respectively.

### Author keyword analysis

This analysis examines the trends and frequencies of author-supplied keywords in allergy research from 1994 to 2025, highlighting key themes and shifts in focus over time.

#### Dominant keywords reflecting central research themes

A total of 6675 “Keywords Plus” and 1741 author-supplied keywords were identified across our data set. Among these, “quality of life” was the most frequently mentioned, appearing 118 times, followed closely by “asthma” with 116 occurrences and “food allergy” with 84 mentions. Other prominent topics included “allergic rhinitis” (69), “children” (67), “omalizumab” (39), “allergy” (35), and “anaphylaxis” (32). Additional keywords such as “health-related quality of life” (29), “atopic dermatitis” (28), and “eczema” (26) highlight the broader themes within allergy research. The data suggest a strong emphasis on both clinical treatments and patient-centered outcomes.

#### Keyword trends and evolving research interests

**Fig 2:**
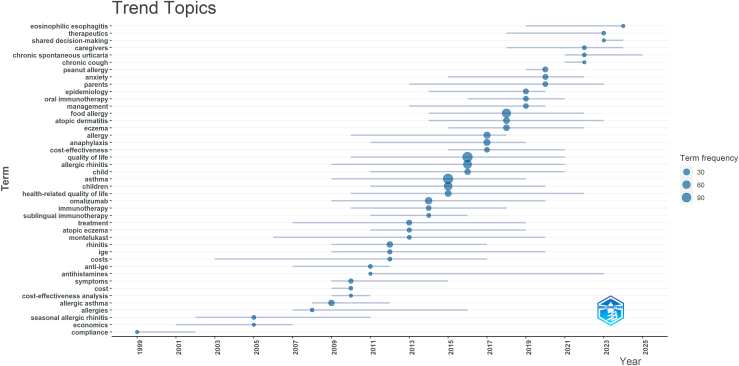
Graphical representation of the most prevalent author keywords in the literature on health/socioeconomic impacts and QoL within the field of allergy. Historical terms such as “chronic urticarial” or “chronic idiopathic urticarial” were mapped to “chronic spontaneous urticaria (CSU)” for consistency with current clinical taxonomy.

The longitudinal analysis of keyword trends reveals dynamic shifts in research focus over time (see Fig 2). Early literature prioritized “compliance” (Q1: 1999), “seasonal allergic rhinitis” (Q1: 2002), and “economics” (Q1: 2001), reflecting initial concerns with the cost and adherence aspects of allergy care. Over time, interest turned toward specific conditions and treatment modalities, with rising mentions of “allergic asthma” (Q1: 2008, Median: 2009), “rhinitis” (Q1: 2009, Median: 2012), and “immunotherapy” (Q1: 2010, Median: 2014), indicating a shift toward targeted therapeutic strategies.

In recent years, the emergence of “oral immunotherapy” (Median: 2019) and “peanut allergy” (Q1: 2019) reflect growing research on innovative, condition-specific interventions. Meanwhile, increased references to “quality of life” (Median: 2016) and “health-related quality of life” (Median: 2015) suggest a shift toward patient-centric metrics and real-world impact. Similarly, the rise in frequency of “atopic dermatitis” (Median: 2018) and “eczema” (Median: 2018) marks a growing attention to skin-related allergic disorders. Emerging themes in the last quarter include “shared decision-making” (Q1: 2023), “caregivers” (Q1: 2018, Q3: 2024), and “chronic spontaneous urticaria (CSU)” (Q1: 2021, Q3: 2025), which reflects the adoption of newer clinical terminology that replaces previous usage of “chronic idiopathic urticaria,” and signals a deeper exploration into personalized care approaches, stakeholder engagement, and chronic allergy conditions.

#### Visual representation of keyword frequencies—Trend topics

Fig 2 presents a visual depiction of these keyword trends across nearly 3 decades, mapping the frequency and publication timelines of major research topics in allergy. The chart reveals distinct phases of focus and emerging priorities.

In the late 2010s, research emphasized disease-specific terms such as “allergic asthma,” “seasonal allergic rhinitis,” and treatments such as “omalizumab” and “sublingual immunotherapy.” The early 2010s saw a shift toward patient-centered outcomes, with increasing focus on “quality of life,” “children,” and “health-related quality of life.” Interest also grew in “food allergy,” “eczema,” and “atopic dermatitis,” highlighting pediatric and dermatological concerns. More recently (2019-2025), psychosocial and chronic care themes, including “anxiety,” “parents,” “caregivers,” and “shared decision-making,” have become more prominent, along with emerging conditions such as “chronic spontaneous urticarial” and “eosinophilic esophagitis.”

### Co-occurrence network of allergy research themes

**Fig 3:**
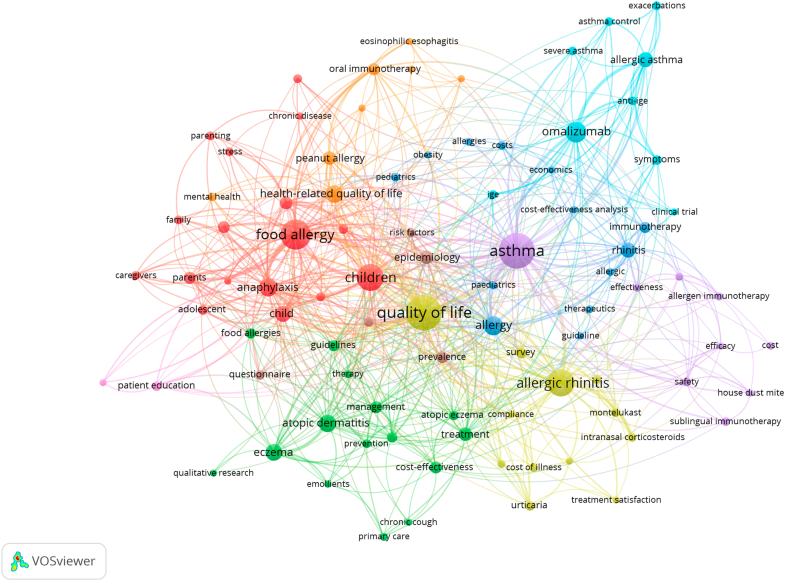
Co-occurrence network visualizations illustrating the interconnected landscape of author keywords within research on health/socioeconomic impacts and QoL in allergy. The term “urticaria” here includes instances of chronic urticaria and chronic spontaneous urticaria (CSU), depending on author keyword use. We note that CSU has only recently emerged as a distinct term in the literature.

The VOSviewer visualization, depicted in Fig 3, maps the co-occurrence of author keywords in allergy research, illustrating the interconnectedness and emphasis of various concepts. The size of each node reflects keyword frequency, whereas the proximity and link strength indicate conceptual relatedness. Central themes such as “asthma,” “quality of life,” and “food allergy” dominate the network, illustrating their foundational role in the literature. These are closely linked with “children,” “caregivers,” and “health-related quality of life,” emphasizing the strong pediatric and patient-centered focus in allergy research.

Clustered domains reveal thematic concentrations:

• The *red* cluster centers on food allergy, encompassing terms such as “anaphylaxis,” “peanut allergy,” and “mental health,” highlighting the psychosocial and clinical challenges of food-induced allergic reactions.
• The *green* cluster is driven by atopic dermatitis and eczema, connecting with treatment, management, and qualitative research, suggesting an emphasis on real-world care and chronic skin conditions.
• The *blue* and *turquoise* clusters bring together asthma, allergic asthma, and pharmacological terms such as “omalizumab,” “anti-IgE,” and “clinical trials,” pointing to advances in biologic treatments and evidence-based practice.
• The *yellow* cluster aligns with allergic rhinitis, paired with keywords such as “intranasal corticosteroids,” “cost-effectiveness,” and “treatment satisfaction,” highlighting therapeutic optimization and economic considerations.

The visualization also reflects cross-cutting concerns such as “cost-effectiveness analysis,” “survey,” “guidelines,” and “epidemiology,” indicating the integration of health economics, clinical standards, and population-level insights. Psychosocial dimensions such as “anxiety,” “stress,” and “parenting” further suggest growing attention to emotional and familial impacts of allergy.

### Most relevant sources publishing in allergy research

**Fig 4:**
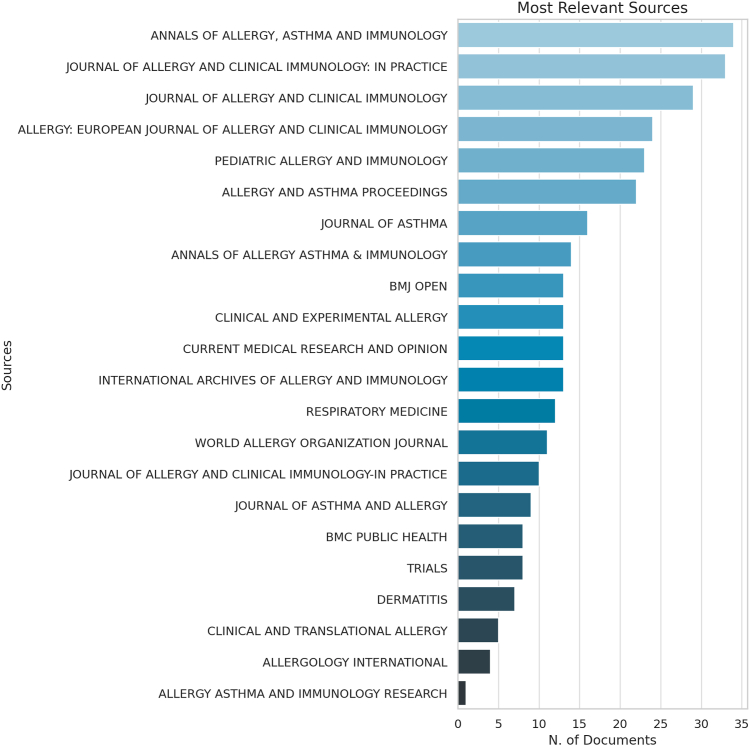
Distribution of documents across journals ranked by the number of publications, focusing on health, socioeconomic impacts, and QoL in allergy research.

This subsection highlights the key journals driving allergy research, showcasing where foundational and innovative studies are published. Based on the bibliometric analysis presented in Fig 4, the *Annals of Allergy, Asthma and Immunology* leads with 34 articles, reaffirming its central role in disseminating foundational and emerging studies. Close behind is the *Journal of Allergy and Clinical Immunology: In Practice* (33 articles), reflecting its emphasis on translating research into clinical settings. The *Journal of Allergy and Clinical Immunology* follows with 29 articles. Other key journals include *Pediatric Allergy and Immunology* (23 articles) and *Allergy and Asthma Proceedings* (22 articles), which emphasize clinical and pediatric dimensions, whereas the *Allergy: European Journal of Allergy and Clinical Immunology* (24 articles) highlights the international breadth of the field. Additional notable sources include the *Journal of Asthma* (16), *BMJ Open* (13), and *Clinical and Experimental Allergy* (13), offering diverse perspectives ranging from clinical trials to open-access research and health economics. In recent years, a marked increase in publication outputs has been observed from *Clinical and Translational Allergy*, *Allergology International*, *Annals of Allergy, Asthma and Immunology*, and the *Journal of Allergy and Clinical Immunology: In Practice*, reflecting the growing interest in clinically actionable allergy research and translational science (Fig 4).

### Global impact and collaboration in allergy research

**Fig 5:**
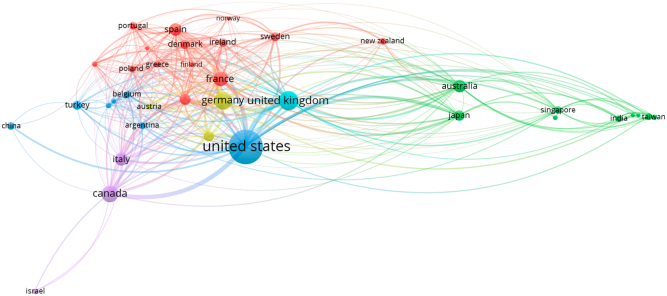
Coauthorship network by country in allergy research.

Fig 5 showcases the global coauthorship network in allergy research, highlighting patterns of international collaboration based on author affiliations. The United States stands out as the most prominent contributor, serving as a central hub with widespread partnerships and a high volume of coauthored publications. Its extensive connections reflect not only research productivity but also a leadership role in fostering cross-border scientific collaboration.

The United Kingdom also demonstrates a strong global footprint, forming robust coauthorship ties with both European and non-European countries. Other influential contributors include Germany, Canada, Italy, France, and Australia, all of which show dense interlinkages, indicating their active engagement in collaborative allergy research. Regional clusters are evident in the network, with European countries forming a tightly connected group, and Asia-Pacific nations, notably Japan, Australia, India, and Singapore, exhibiting strong intraregional and interregional partnerships. Countries such as Turkey, China, and Israel are also integrated into the global research network, contributing to the diversity and depth of international cooperation. Overall, the coauthorship network in Fig 5 illuminates the global and cooperative nature of allergy research, emphasizing how shared challenges in allergy and immunology have led to a highly interconnected and internationally engaged scientific community.

### Strategic thematic evolution in allergy research

**Fig 6:**
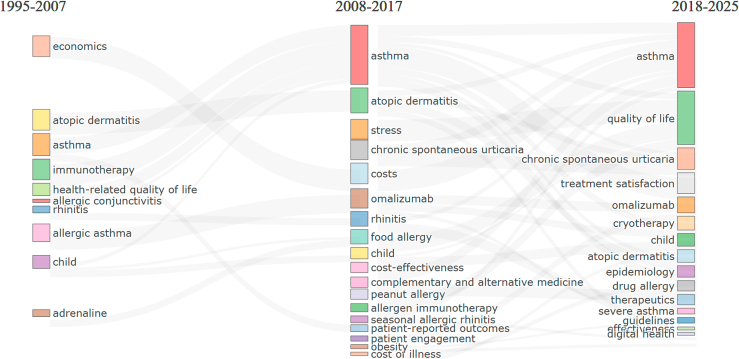
Thematic evolution of key research themes in allergy based on author keyword co-occurrence (1995-2025).

Fig 6 illustrates the temporal progression and thematic transitions in allergy research from 1995 to 2025. The Sankey diagram connects keywords across 3 distinct periods—1995-2007, 2008-2017, and 2018-2025—highlighting how research priorities have evolved and how certain topics have persisted, merged, or diverged over time.

In the early period (1995-2007), research emphasized themes such as “economics,” “allergic asthma,” and “immunotherapy,” reflecting foundational and cost-focused concerns. By 2008-2017, attention had shifted to disease-specific and therapeutic keywords such as “asthma,” “omalizumab,” “food allergy,” and “cost-effectiveness.” In the most recent period (2018-2025), patient-centered areas such as “quality of life,” “treatment satisfaction,” and “digital health” have gained prominence. Themes such as “asthma,” “atopic dermatitis,” and “child” remain important. This visual evolution highlights a broader trend in the field—from clinical efficacy and economic evaluation to holistic, outcome-based, and technology-driven approaches. The diagram provides a comprehensive overview of how allergy research themes are interconnected across time, offering insight into both enduring pillars and emerging frontiers in the discipline.

## Discussion

Our comprehensive bibliometric analysis from 1994 to 2025 highlights the multifaceted nature of allergy research, with 913 documents underlining the scientific community’s ongoing engagement. The average of 41.85 citations per document indicates the high level of interest and significant impact of these scholarly contributions, confirming their foundational role in the broader scientific narrative of allergy. The consistent growth rate in allergy research aligns with the rising prevalence of allergies and the increased recognition of their socioeconomic and QoL implications. Post-2007, there has been a notable surge in related publications, peaking in 2017. This trend is supported by literature such as Warren et al^14^ and Tepler et al,^15^ which explore the diverse socioeconomic impacts of food allergies.

The authorship pattern, averaging 9.03 coauthors per document with a 24.42% rate of international coauthorship, reflects a robust culture of collaboration. This also perhaps highlights the complex nature of allergy research, necessitating an interdisciplinary and global approach to advance understanding and treatment. Most authors are concentrated within clinical medicine, immunology, and public health. However, there is limited representation from social sciences, behavioral psychology, health economics, and digital health disciplines. These areas are critical for advancing patient-centered care models, understanding psychosocial burden, and supporting implementation of scalable, cost-effective interventions. Future research would benefit from actively engaging scholars from these fields to broaden the scope and impact of allergy-related studies.

Longitudinal thematic analysis reveals that although “asthma” continues to be a dominant and persistent theme across all time periods, recent years have seen a notable shift toward “quality of life,” reflecting a growing emphasis on patient-centered outcomes. The emergence of “treatment satisfaction,” “cryotherapy,” and “digital health” as newer themes between 2018 and 2025 indicates an expanding interest in innovative therapies and technology-enabled care models. In addition, themes such as “chronic spontaneous urticaria (CSU)” and “drug allergy” suggest a diversification of research into more specific conditions. CSU has emerged in recent years as a more precise term replacing “chronic idiopathic urticaria,” highlighting increased clinical and terminological granularity in allergy research. The continued presence of “child” and “atopic dermatitis” highlights the sustained interest in pediatric allergy and skin allergy. This thematic evolution demonstrates the field’s adaptive response to emerging clinical needs, evolving patient expectations, and advancements in precision medicine and digital tools. Dierick et al^16^ highlight the need for diverse and targeted research approaches to address the varied impacts of allergic diseases.

Addressing health disparities is essential, as noted by Warren et al,^14^ who emphasize the disproportionate burden of food allergies among minority groups. Schyllert et al^17^ also point out the influence of low socioeconomic status on allergic diseases, particularly among women, highlighting the need for tailored interventions and policies. Economic considerations in allergy research have gained prominence, reflecting Lee and Weiss’s^18^ emphasis on integrating health economics into decision making. The high costs associated with biologics, highlighted by Azzano et al,^19^ necessitate careful resource allocation in allergy clinics.

Longitudinal studies on QoL measures, such as those by Flokstra-de Blok et al,^20^ underline the importance of using both generic and disease-specific questionnaires to fully capture the impact of food allergies on patients’ lives. This dual approach provides a more nuanced understanding of how allergies affect social and psychological domains of health beyond clinical symptoms. Although our bibliometric analysis showcases growing interest and academic vigor in themes related to socioeconomic and QoL impacts of allergy, it also highlights the need for wider collaborations to better investigate this global problem. To foster innovation and knowledge exchange, future studies must actively include underrepresented disciplines such as behavioral science, health informatics, policy research, and education. There is a clear imperative for interdisciplinary collaboration that extends beyond traditional research boundaries, incorporating economic evaluations and QoL measures to develop comprehensive, patient-centered health care solutions.

### Future recommendations

Given the strategic thematic evolution highlighted in the analysis, several key recommendations emerge:

• *Enhanced Interdisciplinary Collaboration*: The intersectionality of allergy research with economic, psychological, and sociological disciplines underlines the need for a collaborative approach that leverages diverse academic perspectives.
• *Economic and QoL Evaluations*: Future studies should embed economic evaluations and QoL assessments within their frameworks to provide a holistic view of the allergy burden. This includes not only cost-effectiveness studies but also analyses of access, equity, and the long-term societal impact of allergic diseases.
• *Diversity in Research Participation*: As disparities in allergy outcomes are evident across socioeconomic strata, concerted efforts must be made to include underrepresented populations in clinical research, in alignment with Warren et al.^21^ Targeted recruitment strategies and culturally responsive research designs are needed to improve inclusivity.
• *Policy and Practice Alignment*: There is a critical need for research to inform policy and clinical practice, ensuring that interventions are evidence-based and grounded in the socioeconomic realities of diverse populations. Greater involvement of public policy scholars and health service researchers can bridge the gap between academic insights and real-world implementation.
• *Longitudinal and Real-world Evidence Studies*: Considering the dynamic nature of allergy research, long-term studies that capture real-world evidence would be invaluable in understanding the changing patterns of allergies and their management.
• *Global Collaboration and Knowledge Exchange*: Building upon the global impact illustrated in the research, international collaborations should be strengthened, enabling knowledge exchange and fostering innovations that can be adapted to local contexts. This includes forming interdisciplinary, multinational research consortia that include voices from low- and middle-income countries.
• *Public Health and Advocacy*: Researchers must also engage in public health advocacy, promoting awareness of allergies and their socioeconomic impact, while advocating for policies that support research funding and patient care.

Thus, our bibliometric analysis highlights the historical trajectory of allergy research and provides a foundation for future inquiry. Integrating economic and QoL dimensions into patient care can lead to a broader understanding of impact of allergies on individuals and their families. This, in turn, can lead to better management of allergic diseases, ultimately enhancing patient care and public health outcomes.

### Study limitations

The scope of our study is confined to the selection of the Web of Science and Scopus databases, which may result in the omission of pertinent literature from other sources. Although this limitation may impact our results, it is unlikely to alter the main trends described in this article. Our keyword-based search strategy, though intentionally designed to include broad and commonly used allergy-related terminology (eg, *allergic asthma* and *omalizumab*), may have inadvertently excluded important but less frequently tagged terms such as *severe asthma*, *benralizumab*, *dupilumab*, and *mepolizumab*. These omissions reflect both limitations in the original indexing practices within the databases and the challenge of balancing search sensitivity with specificity. For instance, *allergic asthma* was included because of its immunologic alignment with IgE-mediated allergic pathways, whereas *severe asthma*, which encompasses a broader clinical phenotype not exclusive to allergy, was not directly captured in our final co-occurrence networks. Similarly, *omalizumab* appeared more frequently than other biologics in the author keywords and was thus visualized more prominently in our thematic maps. Although this reflects historical usage patterns and publication volumes, it does not imply exclusion of the relevance of other biologics in allergy management. Future bibliometric studies might benefit from customized thesaurus-driven text mining or manual curation to capture such nuanced therapeutic entities more comprehensively.

In addition, data cleaning processes, essential for reducing inconsistencies, could introduce discrepancies due to the diverse linguistic use in academic discourse. For instance, recognizing “… University” and “… University Hospital” as separate institutions reflects technical limitations that prevented full data alignment. Despite efforts to correct such issues, manual adjustments could have compromised the accuracy of other data, such as the average publication year, and so the original results were retained. In addition, verification of prolific authors is subject to potential inaccuracies from name spelling variations or institutional changes, highlighting the inherent challenges of bibliometric analyses. A minor overlap in our data highlights some methodological constraints but has minimal impact on the study’s overall conclusions and identified trends.

This study maps allergy research trends, identifying gaps and guiding future strategies to improve outcomes.

### Conclusion

This study provides a roadmap for ongoing and future research highlighting popular as well as less researched themes aimed at improving outcomes for individuals living with allergies. This bibliometric analysis of 913 publications (1994-2025) highlights dominant and emerging themes in allergy research. “Asthma,” “quality of life,” and “food allergy” remain central, whereas newer topics such as “digital health” and “treatment satisfaction” show a shift toward patient-centered approaches. Most publications originate from the United States, followed by the United Kingdom, Germany, and the Asia-Pacific region, demonstrating strong collaboration efforts. However, increased collaboration with underrepresented regions is needed. The integration of socioeconomic and QoL dimensions reflects the field’s evolution toward more holistic care models. Future research should prioritize interdisciplinary approaches, equity in study populations, and real-world evidence to address the diverse impact of allergic diseases. This study provides a strategic foundation for guiding impactful, inclusive, and globally relevant allergy research in the years ahead.

**Key messages**

• **The evolution of allergy research highlights a growing emphasis on QoL, treatment satisfaction, and patient-centered care, particularly in areas such as food allergy and chronic conditions.**
• **Interdisciplinary collaboration—including underrepresented fields such as health economics, behavioral science, digital health, and policy research—is crucial for advancing comprehensive and equitable allergy care.**

## Disclosure statement

The authors reported that there was no funding associated with the work featured in this article.

Disclosure of potential conflict of interest: The authors declare that they have no relevant conflicts of interest.

Data availability statement: The data that support the findings of this study are available from the corresponding author upon reasonable request.
