# Supplementary material for: Mapping allergy research: A comprehensive visual and bibliometric analysis of socioeconomic and quality-of-life dimensions
Source: J Allergy Clin Immunol Glob. 2025 Aug 12;4(4):100553. doi: 10.1016/j.jacig.2025.100553 (PMC12509739; doi:10.1016/j.jacig.2025.100553)
Supplement: Supplementary Data [file mmc1.docx]

***Search Strings - Web of Science***

TS = (((“cost of illness” OR “quality of life” OR “socioeconomic factors” OR “social adjustment” OR “health-related quality of life” OR “economics” OR “psychological wellbeing”) AND (allerg*)) AND (parent* OR physician* OR “health care provider” OR “patient experience” OR “provider experience”) AND (experiences OR preferences OR “decision making” OR “cost effectiveness” OR “barriers to access” OR “healthcare costs” OR “economic burden” OR “treatment outcomes” OR “health outcomes” OR “patient satisfaction” OR “quality of care” OR “accessibility of care” OR “healthcare utilization” OR “healthcare delivery” OR “patient engagement” OR “provider attitudes” OR “health policy” OR “public health” OR “policy making” OR “healthcare policy”))

***Search Strings – Scopus***

TITLE-ABS-KEY (((“cost of illness” OR “quality of life” OR “socioeconomic factors” OR “social adjustment” OR “health-related quality of life” OR “economics” OR “psychological wellbeing”) AND (allerg*)) AND (parent* OR physician* OR “health care provider” OR “patient experience” OR “provider experience”) AND (experiences OR preferences OR “decision making” OR “cost effectiveness” OR “barriers to access” OR “healthcare costs” OR “economic burden” OR “treatment outcomes” OR “health outcomes” OR “patient satisfaction” OR “quality of care” OR “accessibility of care” OR “healthcare utilization” OR “healthcare delivery” OR “patient engagement” OR “provider attitudes” OR “health policy” OR “public health” OR “policy making” OR “healthcare policy”))
